# Structure and Function of Polysaccharides and Oligosaccharides in Foods

**DOI:** 10.3390/foods12203872

**Published:** 2023-10-23

**Authors:** Tianming Yao, Mengting Ma, Zhongquan Sui

**Affiliations:** 1 Department of Food Science & Technology, School of Agriculture and Biology, Shanghai Jiao Tong University, Shanghai 200240, China; yao132@purdue.edu (T.Y.); mmt9497@sjtu.edu.cn (M.M.); 2 Department of Food Science, Whistler Center for Carbohydrate Research, Purdue University, 745 Agriculture Mall Drive, West Lafayette, IN 47907, USA

## 1. Introduction

Polysaccharides and oligosaccharides are abundantly found in various foods. They play essential roles in determining food texture, quality, and nutritional value. Common dietary polysaccharides include digestible starches and non-digestible fibers, such as cellulose, hemicellulose and various food gums. The inherent complexity of polysaccharide structures, derived from the diversity of plant sources and potential chemical modifications, results in a wide range of different physicochemical properties, making them flexible for various applications in the food industry. Oligosaccharides, a specific type of carbohydrate consisting of three to ten sugar units, are also prevalent in foods such as fruits, vegetables, milk, and honey. They exhibit a wide array of structural compositions, including fructo-, galacto-, and xylo-oligosaccharides, which have been extensively used in food products, especially in infant formulas. Although oligosaccharides have historically received less attention compared to simpler sugars or polysaccharides, recent interest has shifted towards their unique properties, such as their role in sweetening, fat replacement, and their potential benefits for gut health.

However, from the perspective of a food chemist, there is a notable deficiency in systematic investigations providing insights into the correlations between the structure and function of polysaccharides or oligosaccharides. Despite some scattered studies, the chemical characterization of polysaccharides often remains inadequate. Factors like low purification levels and the inherent structural heterogeneity of polysaccharides complicate the current functional studies, impeding the establishment of comprehensive structure–function relationships. It is imperative to recognize that even subtle structural differences in polysaccharides can lead to significant functional differences. To bridge this gap, it is vital to obtain more refined structural information about these carbohydrates to elucidate their functional attributes. The refined structural information includes the details concerning monosaccharide composition, glycosidic linkage profiles, chemical motif attachments and molecular weight distributions. In addition, spatial structural information, such as insights into the branching degree, chain length and helical structure, could be considered in functional investigations. Exploring specific structural characteristics of polysaccharides and oligosaccharides can facilitate a more profound understanding of their potential applications in enhancing food texture, nutritional value, potentials in disease prevention and novel food development.

## 2. High Food Quality Depends on Specific Molecular Structures

Texture and flavor represent fundamental constituents of food palatability, serving as pivotal indicators for assessing the quality of food products. Polysaccharides and oligosaccharides exert substantial value in enhancing the textural attributes of foods as texture modifiers due to their low cost, non-toxicity, bio-accessibility, and sustainability, as well as their health gain effects. In this context, the application of food polysaccharides can substantially affect various physicochemical properties of food, such as hydration, gelation, emulsification, and thickening, among others. These features are intricately linked to the molecular structures. For instance, gelling polysaccharides like agarose and pectin rely on the presence of specific crosslink sites which facilitate the formation of organized intermolecular associative structures, resulting in robust gel matrices [1]. The strength of these networks can be fine-tuned through variations in the molecular weight and the branched chain length of the polysaccharides, thereby impacting textural properties, e.g., the ability of the gel to promptly revert to its original form when external stress is applied. Starch, a staple food ingredient, exhibits distinct pasting and rheological characteristics depending on the internal composition of amylose and amylopectin, consequently influencing attributes such as creaminess, thickness, and chewiness in food products.

Although oligosaccharides are not commonly employed as texture modifiers, they can be utilized in combination with polysaccharides to extend the range of food properties and tailor the thickness of food products. These effects are also closely related to fine structures of oligosaccharides. For example, xylo-oligosaccharides (XOS) with different unit lengths (e.g., xylobiose, xylotriose, and xylotetraose) exhibit disparate rheological behaviors. Commercial XOS syrups have vague structural information due to their mixed molecules, often leading to inconsistency in food production [2]. Hence, to overcome this problem and contribute to the development of high-quality, consistent food products, it is imperative to establish the missing link between the specific molecular structures of polysaccharides and oligosaccharides and their functional properties to enhance food quality.

## 3. Impact of Structural Characteristics on Food Nutritional Values

Digestible polysaccharides (e.g., starch) are essential sources of daily energy for humans. However, the rate at which these carbohydrates are digested plays a critical role in maintaining overall health, and this can be attributed to their structures. Rapid digestion of these energy-dense carbohydrates has been associated with various health concerns, including obesity, hyperglycemia, and even type II diabetes. The natural molecular structures of these carbohydrates, such as amylose content and degree of polymerization (DP), are key factors influencing their digestion speed. The refined structure of amylopectin of starch is vital for its nutritional value. Amylopectin can be categorized into short, long and very long chains, each contributing to distinct clustering structures that impact digestive properties. Additionally, starch–protein and starch–lipid complexes commonly formed in food products further affect the nutritional properties of polysaccharides [3]. Accurate modeling of these complexes requires precise molecular structure information, which is essential for deciphering their potential health benefits. Furthermore, specific oligosaccharide structures, such as maltopentaose, have been found to have physiological modulation effects in vivo, such as stimulating the secretion of gut glucagon-like peptide 1 (GLP-1) and influencing the overall speed of food digestion [4]. This discovery has prompted future research directions to focus on obtaining detailed molecular information about digestible carbohydrates in foods.

Non-digestible polysaccharides (e.g., dietary fibers and resistant starch) and oligosaccharides are extensively studied for their use as dietary fibers that can be fermented by human gut microorganisms. This interdisciplinary field has attracted the attention of scholars from diverse backgrounds. However, investigations lack the involvement of food or polymer chemists and often overlook the structural complexity of the substrates during fermentation, leading to contradictory and non-reproducible findings. The primary governing factor for modulating microbial responses in gut fermentation is the carbon source. Structural information, such as component composition, glycosidic linkages, branches, and molecular weight, are all vital factors that create distinct ecological niches for organisms from different taxa, regulating their metabolic outputs and ultimately influencing host health.

## 4. Emerging Novel Food Developments and Functions Tied to Precise Structural Attributes

High-end food manufacturing is undergoing a transformation with the advent of cutting-edge technologies like 3D printing foods, which can offer precision and customizability to individuals with diverse health and taste preferences. Food-grade polysaccharides and oligosaccharides play a pivotal role as the “ink” for 3D printers, imparting essential properties like structural support and adhesion to food materials [5]. Another area of rising consumer interest falls in low-fat products, which have aroused growing interest regarding fat replacement, a role to which polysaccharides can be well fitted. However, the challenge lies in selecting the appropriate molecules and understanding how their molecular structures influence the fat replacement capabilities. Additionally, specific polysaccharides with health-beneficial attributes can enhance both the nutritional and textural profiles of meat analog products, which is a novel application that demands a comprehensive understanding of the structural details of polysaccharides and oligosaccharides.

Overall, this Special Issue seeks to publish high-quality articles involving refined structural information of polysaccharides and oligosaccharides, elucidating a wide range of their functions in food quality, nutrition, health promotion and novel foods.
